# Curcumin-PLGA based nanocapsule for the fluorescence spectroscopic detection of dopamine

**DOI:** 10.1039/d2ra01679f

**Published:** 2022-10-04

**Authors:** Hanine Zakaria, Riham El Kurdi, Digambara Patra

**Affiliations:** Department of Chemistry, American University of Beirut Beirut Lebanon dp03@aub.edu.lb +961 1365217 +961 1350000 ext. 3985

## Abstract

The main purpose of this paper is to design curcumin loaded PLGA nanocapsules for the selective detection of dopamine using fluorescence spectroscopy. In the present work curcumin loaded PLGA nanocapsules were synthesized using a solid-in-oil-in water (s/o/w) emulsion technique. The prepared nanocapsules were coated with a poly(diallyldimethylammonium)chloride (PDDA) polymer to increase the entrapment of curcumin into the core of PLGA polymer. PLGA-Cur-PDDA nanocapsules were characterized using different microscopic and spectroscopic techniques. Unlike free curcumin, the formed CUR-PLGA-PDDA NCs were established as nanoprobes for the selective detection of dopamine molecules. The selectivity and specificity of nanocapsules toward dopamine was achieved by measuring the fluorescence emission spectra of the NCs in the presence of other interference molecules such as tryptophan, melamine, adenine, *etc.* It was noticed that increasing the concentration of the different molecules had no significant change in the fluorescence signal of the nanocapsules. These results confirm the strong quenching between dopamine and curcumin in the nanocapsules. Hence, this fluorescence emission technique was found to be selective, easy and fast with low cost for the determination of dopamine in a concentration range up to 5 mM with a detection limit equal to 22 nM.

## Abstract 2

The main purpose of this paper is to design curcumin loaded PLGA nanocapsules for the selective detection of dopamine using fluorescence spectroscopy.

## Graphical abstract

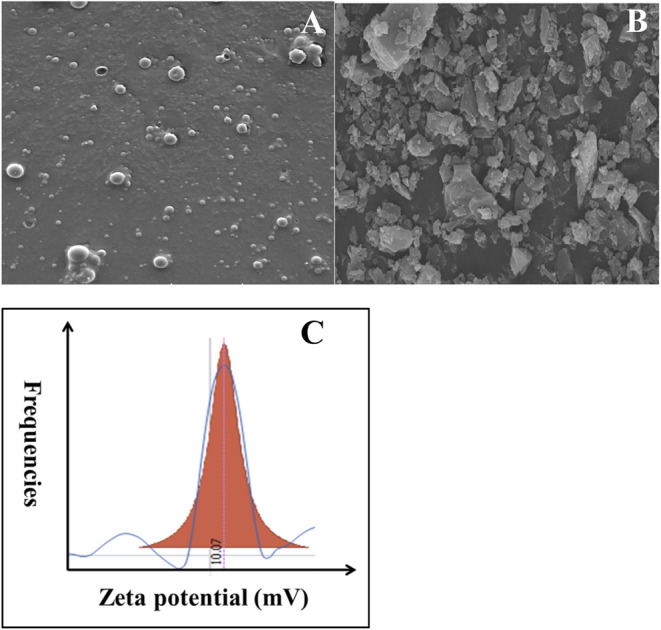

## 1. Introduction

Nanoscience is defined as the study of molecules and structures in the nanometer scale, and the technology that applies it in practical applications is known as the nanotechnology.^1^ Nanocapsules are a characteristic class of nanoparticles made up of two or more active materials characterizing the core and a protective matrix representing the shell.^2^ The size of nanocapsules ranges between 10 and 1000 nm.^3^ Nanocapsules have gained great attention in chemistry, medicine, nanotechnology, materials, and pharmacology due to their wide variety of applications.^4^ The aim of nanocapsule formation is to ensure the entrapment of a specific drug into the membrane of phospholipids, or micelles. The drug encapsulation will help the drug to get through to the exact target.^4^

In fact, curcumin is one of the most used drugs in the biomedical field. Curcumin is a bioactive polyphenol derived from the rhizome of the *Curcuma longa*-a turmeric plant.^5^ Besides its therapeutic benefits such as its antioxidant and anti-inflammatory effects,^6^ antimicrobial and anticarcinogenic activities,^7^ curcumin is being combined to nanomaterials to design effective nanosensors for the detection of specific analytes. For example, liposomal curcumin nanocapsules in the presence of PDDA polymer were used to detect ATP molecule.^8^ In addition, a study conducted by Bechnak *et al.* has proven the efficiency of curcumin/F-108 polymeric nanocapsules in the detection of nucleic acid.^9^ However, curcumin suffers from one major problem which is its poor bioavailability.^6^ Also, other obstacles are associated with curcumin like low water solubility, crystalline nature and alkaline degradation.^10^ Nevertheless, many methods had been studied to enhance the stability and the bioavailability of curcumin like the addition of agents like piperine, and the complexation in novel drug carriers such as liposomes, nanoparticles and micelles.^11^

Poly lactic-*co*-glycolic acid (PLGA) is a copolymer synthesized through a random ring opening copolymerization of the cyclic dimers of the lactic and glycolic acids.^12^ PLGA is a member of the FDA approved biodegradable polymers.^13^ PLGA has been used as a biomaterial because of its biocompatibility and biodegradability.^12^ In addition, PLGA has extensively used in drug delivery applications this due to its mechanical and processing properties.^14^ Also, it possesses a promising application as a platforms for tissue engineering.^13^ PLGA has involved in nanotechnologies and it showed successful diagnostic and therapeutic effects.^12^ Many researches had been done on the incorporation of curcumin into PLGA based nanocapsules. Studies showed that CUR-PLGA nanocapsules have a spherical shape.^15^ Hence, it was confirmed that the loading of curcumin in PLGA could increase the bioavailability of curcumin.^16^ Due to the biodegradable nature of PLGA, it enhances the biocompatibility of curcumin.^17^ Also, the solubility of curcumin is greatly enhanced upon encapsulation.^17^ The antioxidant properties do not alter with long term storage of CUR-PLGA nanocapsules.^18^

Poly(dimethyldiallylammonium chloride) (PDDA) is known as a cationic polyelectrolyte polymer, that can be used as a surfactant in order to increase the stability of the prepared nanocapsules. PDDA is known to be essential in biomedical applications, particularly in drug delivery applications, due to its biocompatibility. It is also easily administered and its method of synthesis is simple and cheap.^19^ Henceforth, PDDA polymer was proven by Othman *et al.*, to be an effective polymer in increasing the stability of curcumin inside DMPC liposomes by decreasing thereby its release.^20^

Dopamine is a catecholamine neurotransmitter in nervous, cardiovascular and hormonal systems, plays an important role as an extracellular chemical messenger.^21,22^ Many diseases result from disorder in the level of dopamine. Low level of dopamine causes sleeping disorders, schizophrenia, Huntington's and Parkinson's diseases.^22^ Whereas, high dopamine level leads to cardiotoxicity which is accompanied with hypertension, drug addiction, increased heart rate and heart failure.^23^ Developing techniques for the detection of dopamine play a significant role in increasing the efficiency of finding suitable treatments. Many methods have been developed for the determination of dopamine including surface plasmon resonance, fluorescence, chemiluminescence, photoelectrochemical sensor, high-performance, liquid chromatography, in addition to electrochemical methods^21,24^ and the calorimetric method.^25^ These methods suffer from low selectivity, the necessity of big amount of samples, long time manipulation, *etc.* For this reason, it was necessary to find a simple method to detect dopamine with high selectivity and sensitivity.

In this work, curcumin molecules were entrapped into PLGA polymer, and coated with poly diallyl dimethyl ammonium chloride (PDDA) polymer in order to increase its stability and bioactivity. Hence, the produced nanocapsules were found to be efficient as fluorescent probe for the sensitive and selective detection of dopamine. The prepared nanocapsules were able to detect dopamine in a wide concentration range till 5 mM.

## 2. Material and methods

### 2.1. Materials

Curcumin (MW: 368.38 g mol^−1^, ≥94%), poly(d,l-lactide-*co*-glycolide) ester terminated (MW: 50 000–75,000), chloroform (MW: 119.38 g mol^−1^, 97%), and poly diallyl dimethyl ammonium chloride (MW: 125.22 g mol^−1^, 20 wt% in H_2_O) were purchased from Sigma-Aldrich. Dopamine was obtained from Acros Organic (MW: 153.18 g mol^−1^). All the chemicals were used as obtained without any further modification.

### 2.2. Preparation of curcumin loaded PLGA nanocapsules CUR-PLGA NCs

CUR-PLGA nanocapsules were prepared using solid-in-oil-in water (s/o/w) emulsion technique.^26^ Briefly, 45 mg of PLGA was socked in 1.5 mL chloroform for 24 hours. Later on, 5 mg of curcumin was added to the mixture and sonicated using probe sonicator for 1 minute. The obtained emulsion was added to 20 mL of poly diallyl dimethyl ammonium chloride (1% w/v) followed by 2 minutes sonication. To evaporate the solvent, the mixture was stirred for 3 hours at 5000 rpm. The obtained emulsion was centrifuged at 15 000 rpm for 10 minutes to precipitate the nanocapsules. The obtained nanocapsules were washed twice with deionized water. Finally, the precipitate was freeze dried in order to obtain CUR-PLGA nanocapsules in form of powder.

### 2.3. Characterization and spectroscopic analysis

JASCO V-570 UV-VIS-NIR spectrophotometer was used to record the absorption spectra at room temperature in a wavelength range of 300–600 nm in a 3 mL cuvette. All fluorescence measurements were carried out at 425 excitation wavelength unless specified. Before freeze drying, the nanocapsules are resuspened in deionized water. From this solution, 0.1 mL was pipetted and diluted into a total volume of 3 mL. Scanning electron microscopy (SEM) analysis was done using a Tescan, Vega 3 LMU with an Oxford EDX detector (Inca XmaW20) at 5 kV accelerating voltage. Briefly, few drops of suspended NCs were deposited on an aluminum stub and coated with carbon conductive adhesive tape. The X-ray diffraction (XRD) analysis was obtained using a Bruker D8 discover X-ray diffractometer equipped with CuKa radiation (*λ* = 1.5405 Å). The monochromator used was a Johansson type monochromator. The X-ray scans were done for 2*θ* between 10° and 45°.

### 2.4. Sample for drug delivery analysis

To minimize the curcumin release form the nanocapsules; 2 different nanocapsules were prepared in the absence and in the presence of poly diallyl dimethyl ammonium chloride. The absorbance of the decanted supernatent obtained after the centrifugation step in the synthesis of the nanocapsules was measured and the mass of free curcumin was calculated.

### 2.5. Sample preparation for dopamine detection

A stock solution of 10 mM of dopamine was prepared by dissolving 15.3 mg in 10 mL of double distilled water. Several solutions were prepared with concentrations in the range of 0 to 5 mM. As per investigating the interference, several concentrations of tryptophan, adenine, uracil, guanine, cytosine, melamine, glutathione, cystine, kreatinine, tyrosine, silymarin were measured at a concentration equal to 5 mM.

Furthermore, the effect of the temperature on the interaction between the nanocapsules and dopamine molecule was studied at 45 °C using a thermostat attached to the spectrofluorometer and with the aid of an external thermometer.

All the experiments were done by keeping the concentration of PLGA-Cur-PDDA NCs constant in a total volume of 3 mL.

## 3. Results and discussion

### 3.1. Characterization of CUR-PLGA-PDDA NCs

**Fig. 1:**
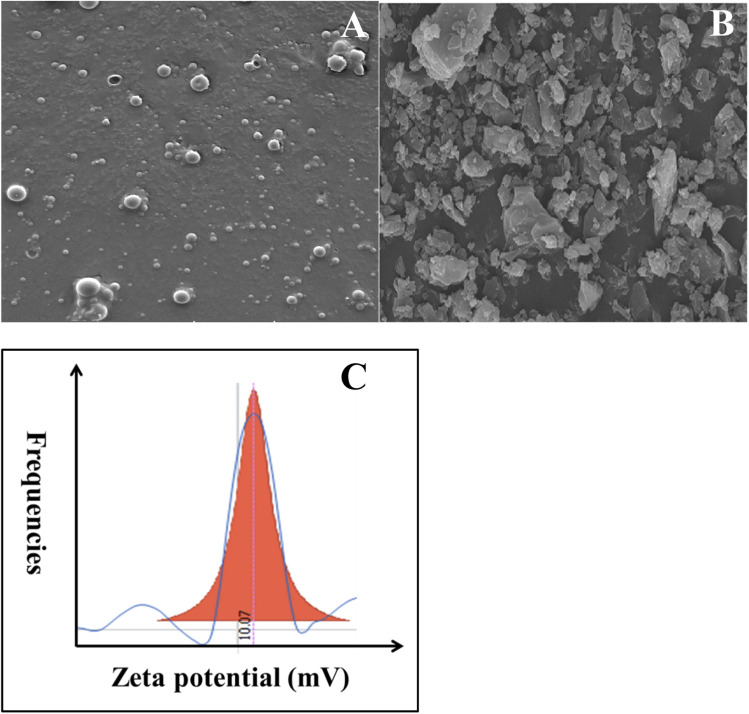
SEM image of (A) CUR-PLGA-PDDA NCs and (B) pure curcumin and (C) zeta potential analysis of CUR-PLGA-PDDA NCs.

Curcumin loaded PLGA nanocapsules were successfully synthesized by solid-in-oil-in water (s/o/w) emulsion technique. This method relays on the fact that the active ingredient (curcumin) is encapsulated as a solid and added to an oil phase (PLGA in chloroform), which formed a solid–oil dispersion. Therefore, in order to enhance the drug absorption, the dispersion is mixed with water to form a continuous phase.^27^ In the beginning, the formed NCs were characterized using SEM and compared with free curcumin. As depicted in Fig. 1A, the nanocapsules were present in spherical shape. However, CUR alone (aggregated solid form) has a rod-like structure (see Fig. 1B). Thus the change in morphology from rod-like to spherical shape indicates and ensures the formation of nanocapsules. Henceforward, the formed nanocapsules were obtained in the size range between 50 and 80 nm.

Furthermore, the surface charge of CUR-PLGA-PDDA nanocapsule was investigated through zeta potential analysis. Interistingly, the formed nanocapsules were positively charged with a value equal to + 10.07 mV (see Fig. 1C). This is mainly due to the presence of ammonium NH_4_^+^ in the PDDA molecules which was adsorbed on the outer layer of the CUR-PLGA NCs causing their stabilization and thus leading to the production of positively charged CUR-PLGA-PDDA NCs.

**Fig. 2:**
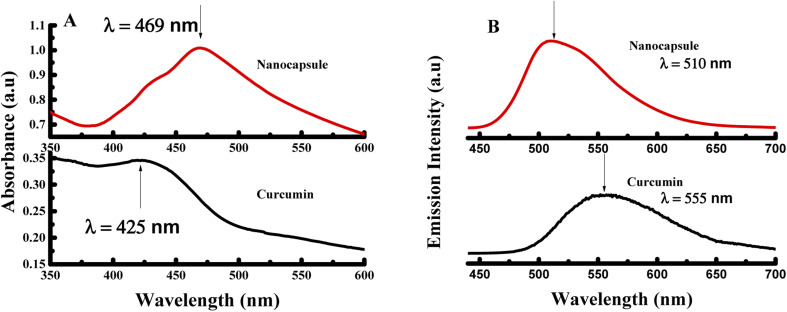
(A) UV-visible spectra and (B) fluorescence spectra of pure curcumin and CUR-PLGA-PDDA NCs excited at *λ*_ex_ = 425 nm.

Moreover, the UV-visible spectrum was established for curcumin and the produced nanocapsules as shown in Fig. 2A. Curcumin absorbs prominently in the UV-visible region around 426 nm (S_0_ → S_1_ transition). However, absorption of CUR-PLGA-PDDA NCs appears at ∼469 nm. Thus, the identification of absorption ∼469 nm makes it easier for establishing the formation of CUR-PLGA-PDDA NCs in the solution. In addition, it is remarkable that a sharp and strong absorption peak was obtained for CUR-PLGA-PDDA NCs. Meaning that, all the curcumin was encapsulated into the core shell of the PLGA polymer, thereby the absence of free curcumin in the formed NCs. Furthermore, the successful production of nanocapsules was verified through the fluorescence emission spectra. As shown in Fig. 2B, a blue shift was occurred when curcumin is being entrapped in the core shell of the PLGA polymer. In fact, the nanocapsules emits at *λ* = 510 nm and curcumin emits at *λ* = 555 nm. This shift is due to the incorporation of curcumin inside the PLGA forming smaller nanocapsules. Also, this can be related to the hydrophobic environment caused by the PLGA polymer.

**Fig. 3:**
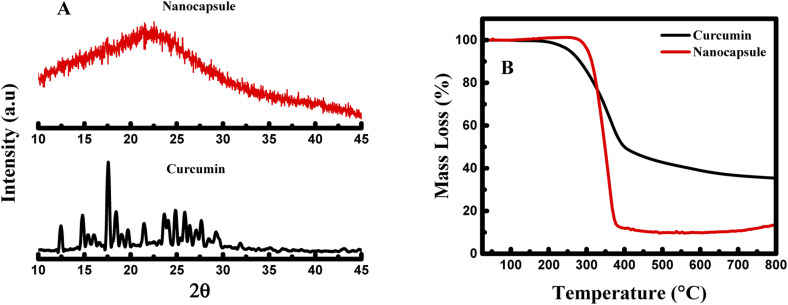
(A) X-ray diffractogram and (B) thermogravimetric analysis of pure curcumin and CUR-PLGA-PDDA NCs.

To further establish the physical characteristic of the nanocapsules, CUR-PLGA-PDDA NCs and curcumin were analyzed by X-Ray Diffraction (XRD) technique. The diffractograms of curcumin and nanocapsules are illustrated in Fig. 3A. The main characteristic peaks of curcumin appeared at diffraction angles of 2*θ* equal to 8.06°, 9.20°, 12.46°, 14.95°, 17.75°, 19.8°, 23.7°, 24.6°, and 26.5° revealing the crystalline form of curcmin.^28^ Yet, as it is shown in the diffractogram of the nanocapsules, these peaks were completely absent. Hence, this confirms the encapsulation of curcumin inside the core shell of the PLGA polymer, inducing the formation of amorphous nanocapsules. Finally, thermogravimetric analysis was performed to assess the stability of the prepared nanocapsules. As shown in Fig. 3B, around 200 °C the weight loss pattern of raw curcumin occurs, where it loses around 65% of its mass between 200 and 560 °C. Hence, a gradual decrease in the mass of curcumin was obtained within the increase of the temperature. Consequently, for CUR-PLGA-PDDA NCs no weight loss was observed around 100 °C, this assures that the synthesized NCs are dehydrated. However, the weight loss of CUR-PLGA-PDDA NCs was observed around 300 °C, after which it shows a sharp weight loss that ends at ∼400 °C. In this temperature range, 86% weight loss was observed. This difference in the degradation pattern means that the temperature over which curcumin is stable has been increased. Therefore, curcumin gained extra stability when encapsulated inside the nanocapsules. Same degradation pattern of PLGA was obtained with Su *et al.*^29^ Accordingly, we can conclude that CUR-PLGA-PDDA NCs follows the same degradation pattern of PLGA.

### 3.2. Encapsulation efficiency and drug delivery study

The encapsulation efficiency of curcumin was calculated based on the beer lambert law. In this case, after centrifugation, the supernatant was collected, and the absorbance was measured. The mass of the free curcumin was obtained based on the absorbance by adopting Beer Lambert's law as below:
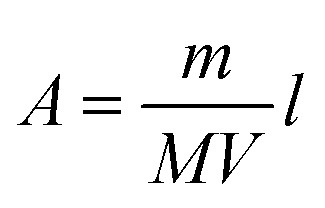
where *A* is the absorbance of the supernatant, *m* is the mass of free curcumin in g, *V* is the volume of the supernatant in mL, *M* is the molar mass in g mol^−1^, *ε* is the molar extinction coefficient of curcumin in M^−1^ cm^−1^, and *l* is the optical path length in cm. Thereby, the encapsulation efficiency (EE) was calculated based on the below formula:
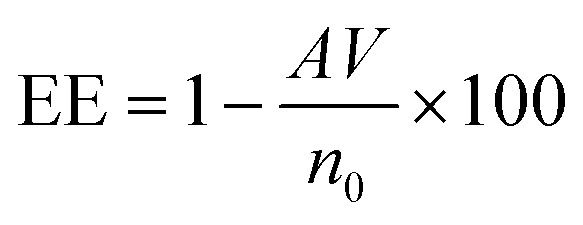
where *V* is the volume of the solution in mL and *n*_0_ is the initial number of mole of curcumin in moles. The molar extinction coefficient was calculated based on the calibration curve of curcumin at pH 7.

In fact, in the absence of PDDA the encapsulation efficiency was equal to 75%. This value had increased to 91% when adding the PDDA layer on the surface of the PLGA-Cur NCs. The enhancement of the EE percentage is due to the increase in the layer added, where it boosts the encapsulation of curcumin into the core of PLGA polymer. The high encapsulation efficiency value obtained in our case was similar to the EE value calculated by Gao *et al.*^30^

**Fig. 4:**
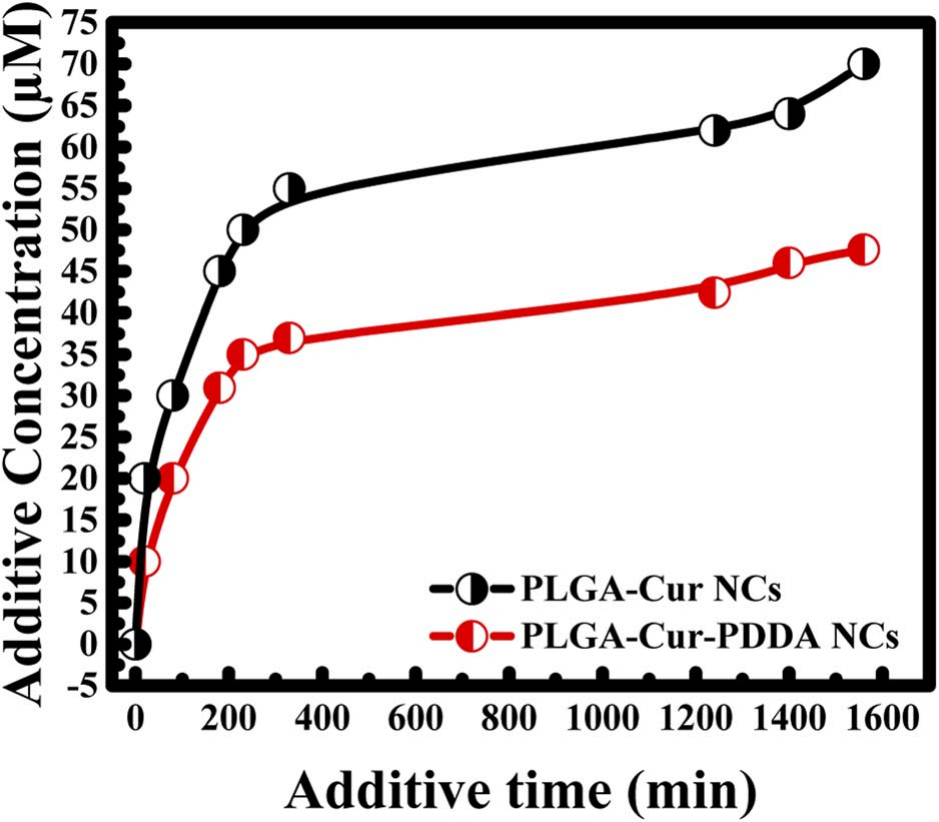
Effect of PDDA layer coated on the surface of the PLGA-Cur NCs in curcumin release.

Furthermore, the drug release for both nanocapsules was investigated. For this, in the beginning a given amount of nanocapsules was kept for a specific time in given volume of double distilled water, then separated by centrifugation and concentration of the supernatant was measured. In the next step, again a given volume of double distilled water was added to the pallet of nanocapsules and kept for another specific time followed by centrifugation to measure concentration. The procedure was repeated till saturation in curcumin release was obtained. To find out total amount of curcumin release for a particular time interval, additive concentration and additive time were estimated by adding concentration and time from the previous measurements. Thus, additive concentration, total concentration curcumin release, increased with time. Interestingly, it was found that faster release of the drug is obtained when the nanocapsules surface was not coated by any PDDA layer (see Fig. 4). However, the rate of release decreases by adding PDDA layers. Such layer by layer assembly improves the stability of the nanocapsules.^31^

### 3.3. Detection of dopamine through fluorescence emission analysis

Nanomaterials are well recognized to own outstanding electrical, optical, thermal, catalytic properties and strong mechanical strength, which offer great chances to build nanomaterials-based probes.^32^ Based on these facts, the formed CUR-PLGA-PDDA NCs were used as nanoprobe for the detection of dopamine.

**Fig. 5:**
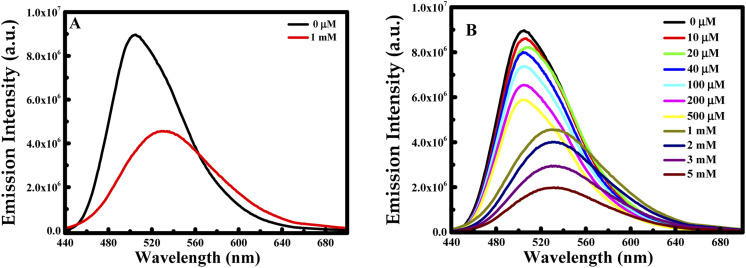
Emission intensity at *λ*_ex_ = 425 nm of CUR-PLGA-PDDA NCs in the presence of (A) 1 mM of dopamine and (B) several concentration of dopamine in the range of 10 μM to 5 mM at 25 °C.

First of all, CUR-PLGA-PDDA NCs were mixed with 1 mM of dopamine and the emission intensity of was measured. Interestingly, a remarkable decrease was noticed accompanied with a red shift (see Fig. 5A). Based on this, several solutions were prepared with different dopamine's concentration in the range from 10 μM to 5 mM. As presented in Fig. 5B, the emission intensity of the nanocapsules decreases within the increase in dopamine's concentration. However, at high concentration of dopamine, higher than 1 mM, a remarkable red shift occurred from 504 nm to 531 nm, meaning that curcumin is being presented in the polar region. Thus, when varying the concentration from 10 to 500 μM, the emission intensity decreases slightly. This decrease can be due to the binding of the negatively charged dopamine, to the positively charged NCs. However, the enhancement of dopamine's concentration leads to an effective fluorescence quenching because at higher concentration of quencher normally quenching sphere action is operative. This is because of fact that nanocapsules helps to bring dopamine closer to curcumin encapsulated in the nanocapsules.

During fluorescence intensity reduction, the quenching efficiency can be symbolized as (*I*_0_/*I*)/*I*_0_, where *I*_0_ and *I* represent the fluorescence intensity of curcumin in the NCs in the absence and presence of dopamine. Hence, the quenching constant (*K*_sv_) can be found using Stern–Volmer equation:*I*_0_/*I* = *K*_sv_ × *C*_dopamine_ + 1

**Fig. 6:**
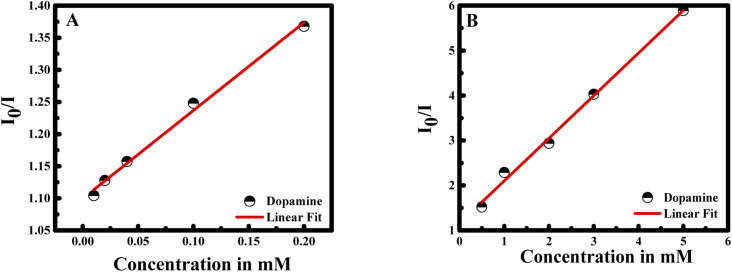
Linear correlation of *I*_0_/*I* of CUR-PLGA-PDDA *vs.* concentration of dopamine (A) from 0.01 to 0.2 mM; and (B) from 0.5 to 5 mM.

The *I*_0_/*I* at the maximum wavelength was plotted *versus* the concentration of dopamine as shown in Fig. 6A and B. The *I*_0_/*I* ratio signal is linear to dopamine concentration in the ranges 0.01–0.2 and 0.5–5 mM. The linear equations for the two concentration ranges are *I*_0_/*I* = 1.37047[dopamine] + 1.09967 with a correlation coefficient of 0.99726 and *I*_0_/*I* = 0.94765[dopamine] + 1.15409 with a correlation coefficient of 0.99484. The limit of detection is found to be 22 nM referring to 3*σ*/*s* criteria, where *σ* is the standard deviation of the measurements and *s* is the slope of the calibration curve.^31^ The efficiency of the method was compared to previous study in the literature (see Table 1).

**Table 1:** Method used to detect dopamine

Method used	Concentration range	LOD	Interference
FRET between acridine orange and CuO NPs^32^	1–40 μM	40 nM	Ascorbic acid, uric acid, glucose, tryptophan and acetaminophen
A simple and convenient fluorescent strategy based on graphene quantum dots^33^	0.5–120 μM	0.16 μM	NaCl, KCl, CaCl_2_, glucose, cysteine, ascorbic acid, epinephrine
Calorimetric detection using microfluidic paper^34^	0.5–4.75 μM	0.37 μM	Ascorbic acid, uric acid
Calorimetric detection using gold nanoparticles^35^	0.5–10 μM	1.85 μM	Amino acids, glucose, ascorbic acid, uric acid
FRET between PLGA-Cur-PDDA (our work)	0.01–0.2 mM 0.5–5 mM	22 nM 1.89 μM	Tryptophan, adenine, uracil, guanine, cytosine, melamine, glutathione, cystine, kreatinine, tyrosine, silymarin, ascorbic acid, uric acid, l-tyrosine, tymarine, l-dopamine

In this case, the plot between *I*_0_/*I* and the concentration of dopamine showed a good linear relationship (with *R*^2^ = 0.9951) in a wide concentration range from 0 to 5 mM. Moreover, the Stern–Volmer equation can be fitted as: *I*_0_/*I* = 0.94765 × *C*_dopamine_ + 1, while *K*_sv_ was found to be 0.94765 mM, equal to the slope value of the linear fit. Consequently, the *K*_sv_ value is considered large which confirms the binding between dopamine and curcumin.

**Fig. 7:**
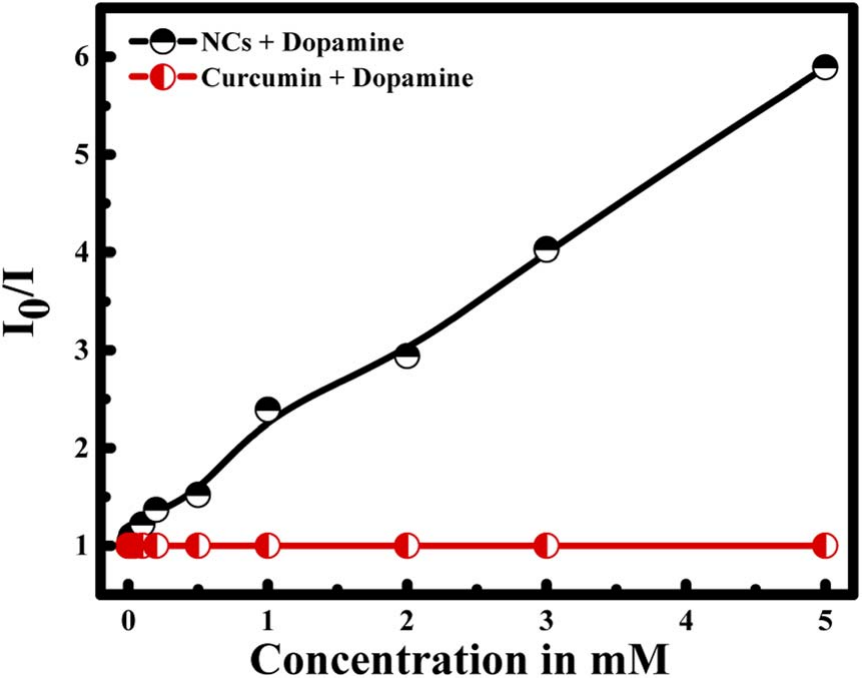
Selectivity of CUR-PLGA-PDDA NCs for the detection of dopamine compared to free curcumin.

To prove the efficiency and the role of the nanocapsule, a control experiment was handled in the presence of curcumin alone and dopamine in aqueous solution. Interestingly, *I*_0_/*I* of curcumin remain constant in the presence of different dopamine's concentration (see Fig. 7). These results reveal the role of the encapsulation of curcumin into the core of PLGA polymer in the presence of PDDA layer. In fact, the negatively charged curcumin cannot interact with the dopamine having also a negative surface charge. Hence, the presence of PDDA polymer increases the surface charge of the nanocapsule; and consequently, facilitates the interaction between dopamine and the nanocapsules. Thus, bringing dopamine and curcumin close enough to have fluorescence quenching.

**Fig. 8:**
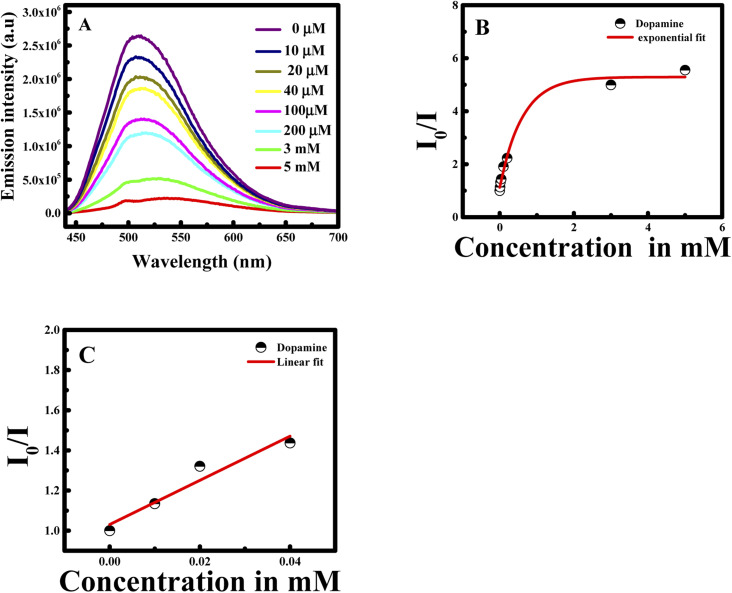
(A) Emission intensity of CUR-PLGA-PDDA NCs in the presence of different dopamine concentration at *λ*_ex_ = 425 nm; (B) correlation of *I*_0_/*I* of CUR-PLGA-PDDA *vs.* concentration of dopamine at 45 °C; and (C) linear correlation of *I*_0_/*I* of CUR-PLGA-PDDA *vs.* concentration of dopamine at 45 °C.

**Fig. 9:**
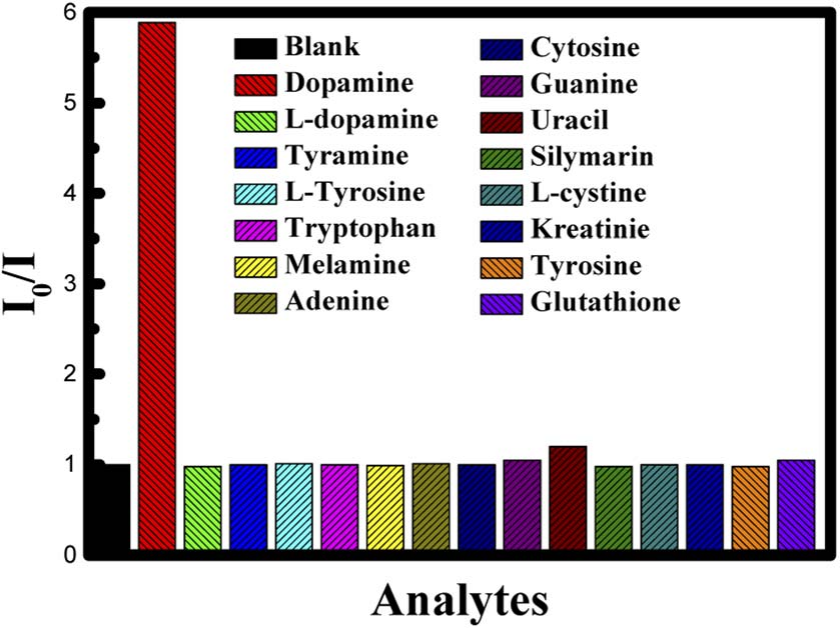
Ratio of emission intensity (*I*_0_/*I*) of CUR-PLGA-PDDA NCs for different species.

To understand the effect of temperature on the interaction between CUR-PLGA-PDDA NCs and dopamine, fluorescence spectra were recorded at an elevated temperature (45 °C). Similar to the previous results, fluorescence intensity showed a decrease with increase in dopamine concentration (see Fig. 8A & B). However, this decrease in fluorescence intensity saturated exponentially as shown in Fig. 8B at higher concentration suggesting a quenching sphere-of-action. Therefore Stern–Volmer equation was applied to lower concentration of dopamine as shown in Fig. 8C. The Stern–Volmer constant was estimated, and it was found that the *K*_sv_ value changed from 0.947656 mM^−1^ to 11.0019 mM^−1^ as temperature increased from room temperature to 45 °C. Temperature effect can tell whether the system is undergoing a static or a dynamic quenching.^33^ The obtained result confirms that static quenching is taking place. The increase in temperature leads to a decrease in the stability of the formed complex, which would decrease the static quenching efficiency.^33^ In contrast, in the present case increase in temperature increased the quenching rate (*K*_sv_) and at higher temperature increase in concentration made quenching sphere-of-action operative. This may be because at higher temperature curcumin is more displaced from the polar to the non-polar region, thus, bringing more dopamine in close contact with the curcumin resulting higher quenching rate. This is further confirmed from the fact that a red shift in fluorescence wavelength maximum was observed only when increasing the concentration of dopamine at 25 °C, this red shift was totally absent when increasing the temperature to 45 °C. Furthermore, the assessment of the selectivity and specificity of the PLGA-Cur-PDDA NCs toward dopamine detection was attained by measuring the fluorescence emission of the NCs in the presence of other interference molecules such as l-tyrosine, tyramine, l-dopamine, tryptophan, melamine, adenine, *etc.* These molecules were selected because they own comparable structures to that of dopamine, so they can typically interfere in dopamine detection. Thus, in Fig. 9 it is obvious that increasing the concentration of the different molecules had no significant change in the fluorescence signal of the nanocapsules. These results confirm the strong quenching between dopamine and curcumin in the nanocapsules.

**Fig. 10:**
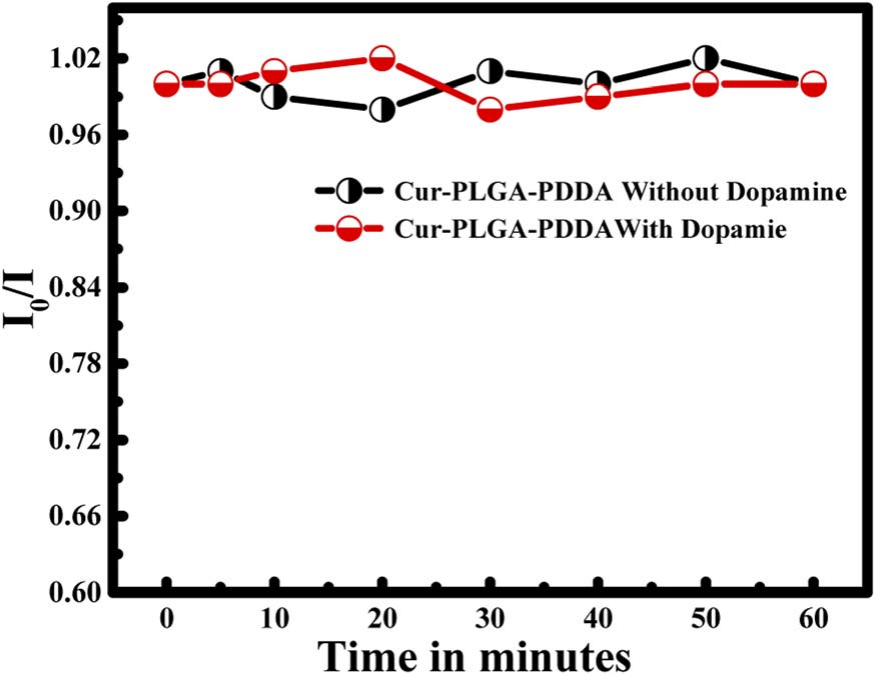
Plot of *I*_0_/*I* of CUR-PLGA-PDDA with time in the absence and presence of dopamine.

Moreover, the stability of the proposed system was done by measuring the fluorescence emission intensity within 1 hour in the absence of dopamine and in the presence of dopamine (*C* = 2 mM). The obtained results are normalized with respect to the ratio obtained at zero time. Hence, within 1 hour *I*_0_/*I* remain constant revealing the stability of the proposed nanoprobe in the detection of dopamine (see Fig. 10).

Finally, to test the applicability, the analytical recovery of three unknown samples was estimated by using the obtained fitted calibration curve. The obtained results where summarized in Table 2. The percent of recovery of dopamine was obtained to be between 98.75 and 100.5% (*n* = 3).

**Table 2:** Recovery percentage of the proposed method

	Theoretical concentration (mM)	Experimental concentration (mM)	Recovery (%)
Sample 1	0.03	0.0298	99.3
Sample 2	0.4	0.402	100.5
Sample 3	4	3.95	98.75

## 4. Conclusion

In summary, curcumin loaded PLGA nanocapsules were successfully coated by poly(diallyldimethylammonium)chloride (PDDA) polymer. The formed PLGA-Cur-PDDA NCs were used to develop a new nanosensing scheme for determination of dopamine. The binding constant was estimated using the Stern–Volmer equation to be around 0.96 mM. This value confirms the binding between dopamine and curcumin, relieving the efficiency of the nanocapsule as nanoprobe to detect dopamine. Based on this method, no interference from other biological and chemical analogues was observed, confirming the selectivity of the proposed nanoprobe. Finally, the method gave a detection limit of 22 nM and works well in the concentration range up to 5 mM.

## Conflicts of interest

There are no conflicts to declare.

## Supplementary Material
